# The Structural, Functional and Evolutionary Impact of Transposable Elements in Eukaryotes

**DOI:** 10.3390/genes12060918

**Published:** 2021-06-15

**Authors:** Dareen Almojil, Yann Bourgeois, Marcin Falis, Imtiyaz Hariyani, Justin Wilcox, Stéphane Boissinot

**Affiliations:** 1 New York University Abu Dhabi, Saadiyat Island, Abu Dhabi P.O. Box 129188, United Arab Emirates; da2451@nyu.edu (D.A.); mjf538@nyu.edu (M.F.); ieh211@nyu.edu (I.H.); jw5478@nyu.edu (J.W.); 2 School of Biological Sciences, University of Portsmouth, Portsmouth, UK; yann.bourgeois@port.ac.uk; 3 Center for Genomics and Systems Biology, New York University Abu Dhabi, Saadiyat Island, Abu Dhabi P.O. Box 129188, United Arab Emirates

**Keywords:** transposable elements, genome evolution, eukaryotes

## Abstract

Transposable elements (TEs) are nearly ubiquitous in eukaryotes. The increase in genomic data, as well as progress in genome annotation and molecular biology techniques, have revealed the vast number of ways mobile elements have impacted the evolution of eukaryotes. In addition to being the main cause of difference in haploid genome size, TEs have affected the overall organization of genomes by accumulating preferentially in some genomic regions, by causing structural rearrangements or by modifying the recombination rate. Although the vast majority of insertions is neutral or deleterious, TEs have been an important source of evolutionary novelties and have played a determinant role in the evolution of fundamental biological processes. TEs have been recruited in the regulation of host genes and are implicated in the evolution of regulatory networks. They have also served as a source of protein-coding sequences or even entire genes. The impact of TEs on eukaryotic evolution is only now being fully appreciated and the role they may play in a number of biological processes, such as speciation and adaptation, remains to be deciphered.

## 1. Introduction 

Since their early discovery by Barbara McClintock, the impact that transposable elements (TEs) may have on their hosts has been a topic of great controversy. Following McClintock’s proposition that TEs may control gene action [1], a number of studies have confirmed an important role of TEs in gene regulation [2,3] and as a source of protein-coding sequences [4], both of which can benefit the host. In contrast, the observations that TEs segregate at low frequency in natural populations of many organisms [5,6,7,8,9,10,11] and that uncontrolled TE amplification can cause hybrid dysgenesis in *Drosophila* [12,13,14,15] suggest a negative impact of TEs on fitness. Another perspective on TEs came from the realization that differences in haploid genome size result from the differential amplification of TEs and are unrelated to the complexity of organisms. This led to the view that TEs may just be parasitic components of genomes and of little evolutionary significance (reviewed in [16]). Advances in genome sequencing, as well as progress in molecular biology methods, shed new light on the impact TEs have on eukaryotic genomes and the picture that has emerged is more nuanced and more complex than anticipated. Although the vast majority of TE insertions are, like other types of mutations, either neutral or deleterious, TEs can also serve as an extraordinary source of evolutionary novelties and have been involved in the evolution of a number of important biological processes across eukaryotes. Here we will review the impact TEs may have on their eukaryotic host in terms of genome size, structural variation, gene and genome regulation, protein-coding evolution, adaptation and speciation, with the goal of contributing to a more holistic understanding of the interactions between TEs and their host.

## 2. TEs in Eukaryotic Genomes

### 2.1. What Are Transposable Elements?

The term “transposable element” covers a large diversity of sequences that have in common their ability to move from one genomic location to another via an active process mediated by an integrase-related enzyme. There are a number of excellent reviews describing in detail the classification of TEs (see for instance [17,18,19,20]) and we present here a brief overview of the diversity of TEs. TEs are typically classified based on their mode of transposition and sequence similarity. Elements that are mobilised via an RNA intermediate are called Class I TEs, and are generally referred to as retrotransposons, while those that propagate as DNA are Class II TEs and are called DNA transposons.

All autonomous Class I elements encode a reverse transcriptase suggesting a common origin [21] and mobilise by a copy-and-paste mechanism. They are further classified based on the presence or absence of Long Terminal Repeats (LTR). In LTR-containing retrotransposons, reverse transcription occurs in the cytoplasm (or possibly in the nucleus [22]) and the resulting cDNA is subsequently integrated into the genomic DNA. This group is widespread in eukaryotes and includes the LTR-retrotransposons sensu stricto (from which infectious and endogenised retroviruses are derived [21]) and the *DIRS* elements [23]. Non-LTR retrotransposons (which include the Long Interspersed Nuclear Elements, or LINEs, and the *Penelope* elements [24]) use a mechanism of transposition called Target Primed Reverse Transcription, in which reverse transcription occurs in the nucleus at the site of insertion [25,26,27]. Despite a strong *Cis* preference [28], the retrotransposition machinery of non-LTR retrotransposons can also act in *Trans* on other transcripts and mediate the transposition of non-autonomous elements, such as the Short INterspersed Elements, or SINEs, which can be derived from tRNA or other non-coding RNAs [29,30,31].

Class II elements include four main categories that are not evolutionarily related and do not have much in common, except that their mode of transposition does not include an RNA intermediate. The most diverse and widespread group of DNA transposons (DDE transposons or TIR transposons depending on the author) have a very simple structure consisting of a single open-reading frame (ORF) encoding for a DDE transposase [32], which recognises short flanking Terminal Inverted Repeats (TIR), cuts the element and inserts it elsewhere in the genome, hence the name cut-and-paste transposons [33]. A second group, called *Cryptons*, also use a cut and paste transposition mechanism, but in this case, the process is affected by a tyrosine recombinase [34]. Although the cut-and-paste mode of transposition involves the excision of a copy and its insertion at a different location, these elements can increase to extremely large numbers if transposition occurs during replication or if the excised site is repaired using homologous recombination with a chromosome still containing the insertion [33]. The *Helitrons* use a rolling-circle replicative mechanism mediated by a protein with an endonuclease and a helicase domain [35,36]. Finally, the *Polintons* or *Mavericks* are very large elements containing up to 20 ORFs [37,38]. They mobilise by a self-synthesizing mechanism involving direct synthesis of a DNA copy by a protein-primed polymerase B. DNA transposons also mediate the amplification of non-autonomous elements that are typically derived from autonomous copies that have suffered internal deletions [39,40,41]. The diversity and number of non-autonomous elements are often far greater than their autonomous relatives.

### 2.2. The Abundance and Diversity of TEs in Eukaryotic Genomes

**Figure 1:**
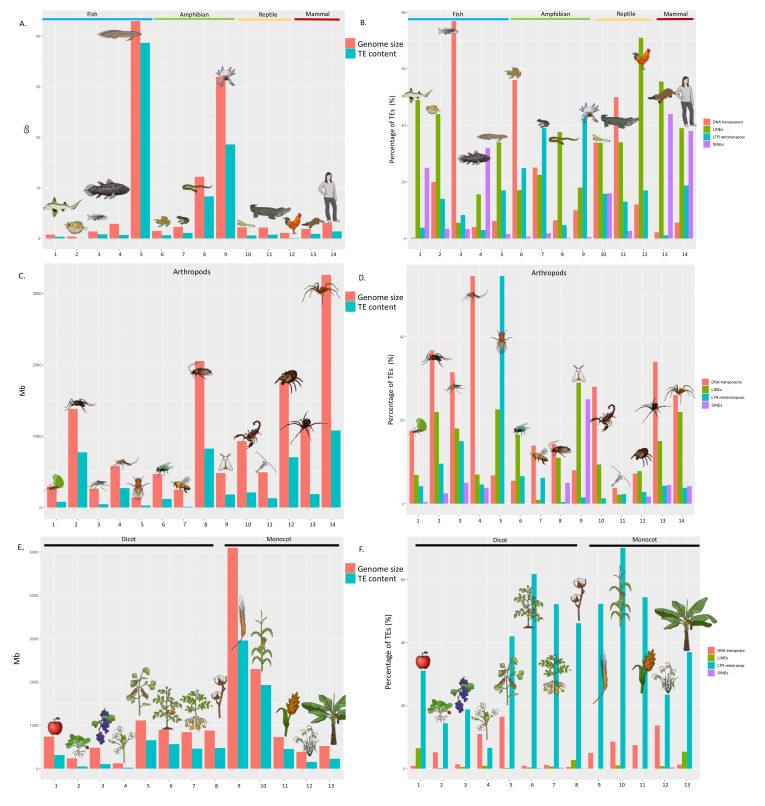
Genome size and its TE content across different organisms. (**A**) Genome size and its TE content in Gb across different vertebrates, from left to right: (1) *Callorhinchus milii*, (2) *Tetraodon nigroviridis*, (3) *Danio rerio*, (4) *Latimeria chalumnae*, (5) *N. forsteri*, (6) *Xenopus tropicalis*, (7) *Nanorana parkeri*, (8) *Ichthyophis bannanicus*, (9) *Ambystoma mexicanum*, (10) *Anolis carolinensis*, (11) *Alligator mississipiensis*, (12) *Gallus gallus*, (13) *Ornithorhynchus anatinus*, and (14) *Homo sapiens*. (**B**) Percentages of TEs types (DNA transposons, LINEs, LTR retrotransposons, and SINEs) of the vertebrates presented in (**A**). (**C**) Genome size and its TE content in Mb across different arthropods, from left to right: (1) *Acromyrmex echinatior*, (2) *Aedes aegypti*, (3) *Anopheles gambiae*, (4) *Culex quinquefasciatus*, (5) *Drosophila melanogaster*, (6) *Lucilia cuprina*, (7) *Apis mellifera*, (8) *Blattella germanica*, (9) *Bombyx mori*, (10) *Centruroides exilicauda*, (11) *Eurytemora affinis*, (12) *Ixodes scapularis*, (13) *Latrodectus Hesperus* and 14) *Loxosceles reclusa*. (**D**) Percentages of TEs types of the arthropods presented in (**C**). (**E**) Genome size and its TE content in Mb across different plants, from left to right: (1) *Malus domestica* (2) *Fragaria vesca*, (3) *Vitus vinifera*, (4) *Arabidopsis thaliana*, (5) *Glycine max*, (6) *Solanum lycopersicum*, (7) *Solanum tuberosum*, (8) *Gossypium raimondii*, (9) *Hordeum vulgare* (10) *Zea mays*, (11) *Sorghum bicolor*, (12) *Oryza sativa* and (13) *Musa acuminate.* (**F**) percentages of TEs types of the plants presented in (**E**). Data from [45,46,56,59,60,61,62,63,64].

Although TEs are nearly ubiquitous in eukaryotes, their abundance differs considerably among species and is largely responsible for differences in haploid genome size [42]. At one extreme, the 2.9 Mbp genome of *Encephalitozoon intestinalis* and the 23 Mbp genome of the malaria parasite *Plasmodium falciparum* are completely devoid of TEs [43,44]. At the other extreme, the 43,000 Mbp genome of the lungfish *Neoceratodus forsteri* is 90% repetitive [45]. Large differences can also be found within eukaryotic lineages, as exemplified in plants, arthropods and vertebrates (Figure 1A,C,E). For instance in insects [46], the 99 Mbp genome of the Antarctic midge *Belgica antarctica* contains less than 1% TEs [47] while 65% of the 6500 Mbp genome of the migratory locust *Locusta migratoria* consists of TEs [48]. Substantial differences can even be found among closely related organisms. For instance, in the genus *Entamoeba*, the genome of *E. histolytica* contains ~20% TEs, while the proportion is ~10% in E. *dispar* [49]. Similarly, in the genus *Oryza*, the genome of the wild rice species *O. australiensis* has more than doubled in size over a 3 my period because of TE amplification resulting in a 965 Mbp genome, while the genome of some of its closest relatives is 375 Mbp [50]. In the fungus *Leptosphaeria maculans*, some strains have experienced a 30% increase in genome size due to TE amplification [51]. In contrast, some lineages exhibit only minor variation in TE content, which may reflect some constraints on genome size or, conversely, relatively high tolerance for genome expansion. Birds, for instance, have a relatively conserved genome size [52] possibly because of the metabolic cost associated with active flight [53], despite the fact that TEs are active in most bird lineages [54,55]. Salamanders, on the other hand, have experienced extreme and independent TE amplification resulting in gigantic genomes, ranging from 14,000 to 120,000 Mbp [56,57,58].

TE diversity also differs considerably among organisms (Figure 1B,D,F). Within vertebrates, fish, amphibian and reptilian genomes typically harbour a large diversity of TEs, with both class I and class II elements [59,65,66], each being represented by a myriad of families, as exemplified by the genome of the green anole *A. carolinensis* [40,67]. In contrast, the genome of most birds and mammals is dominated by a single type of non-LTR retrotransposon *CR1* in bird [54], *L1* in placental mammals [17,66] and *L2* in monotremes [68]. In plants, TE diversity is also variable among species but differences in genome size result mostly from the differential amplification of LTR retrotransposons. Differences in diversity can also be observed at a finer scale and the relative proportion of different types of TEs can evolve rapidly. For instance, in the *Heliconius* butterfly radiation, the relative representation of different categories of TEs has changed markedly [69]. Interestingly, the relationship between TE diversity and abundance is not straightforward. Genome size and TE diversity are positively correlated up to a genome size of 500 Mbp [70], although some very compact genomes harbour very few TE families, such as the baker’s yeast [71]. Genomes that are larger than 500 Mbp tend to have a lower TE diversity due to the amplification of a specific type of element, which accounts for most of the genome mass.

These comparisons of TE content across the eukaryotic tree of life raise the question: why do genomes differ so much in their TE composition and consequently in their genome size? It is easier to understand why some genomes remained small despite the long-term persistence of TE activity. In a compact genome, such as the one of *D. melanogaster*, new euchromatic insertions are more likely to be deleterious and are therefore eliminated by purifying selection, rarely reaching high population frequency or fixation [10]. Because selection against TEs is dependent on the length and copy number of the insertions [72], the more likely cause for the deleterious effect of new insertions results from their ability to mediate ectopic recombination events [10]. It is, however, more difficult to explain the evolutionary increase in genome size, which can result in genome gigantism in some groups. Genetic drift is a process that will counteract the effect of selection and may favour the fixation of slightly deleterious TE insertions. This is supported by the fact that organisms with large effective population size tend to have smaller genomes than organisms with small population size, in which drift is stronger [73]. However, the resulting accumulation of TEs will pose additional problems because the increase in the number of copies increases the chance of ectopic recombination. In addition, if a genome contains a low diversity of TEs but if these TEs are represented by many copies the chance of ectopic recombination will be higher than in a genome with a large diversity of TEs represented by a small number of copies [74]. A possible solution to this problem is the evolution of a lower rate of ectopic recombination in genomes that contain a large number of similar copies, possibly via increased surveillance by the mismatch repair system [74,75]. Since ectopic recombination events can be drivers of DNA loss [76,77,78], a higher rate of DNA loss, via large deletions, is expected in species with a diverse repertoire of TEs represented by small copy numbers (as in most non-mammalian vertebrates) than in genomes containing many similar copies (such as mammals and salamanders). This hypothesis is supported by a faster decay of non-LTR retrotransposon insertions caused by large deletions in reptiles and fish than in mammals [67,79]. Consistent with the hypothesis above, the gigantic genome of some salamanders and caecilian is characterised by an extremely reduced rate of DNA loss and ectopic recombination [56,80,81]. Interestingly, the rate of transposition and the rate of DNA loss can counteract each other perfectly, resulting in the long-term stability in genome size observed in birds and mammals, a process termed the “accordion” model of genome evolution [82].

### 2.3. The Population Dynamics of TEs

The abundance, diversity and genomic distribution of TEs depend on several factors, which include the number of copies produced (the transposition rate) and the number of copies that accumulate in the genome, with the latter depending on population processes such as selection and drift. The impact of TEs on their host’s fitness is a key parameter to understand their population dynamics. TEs are more likely to rise to high frequency in populations and accumulate in the genome if they have little to no deleterious effect. However, the impact of TEs on host fitness is often neutral or negative and depends on (1) whether TEs insert in functional regions and display regulatory features that affect nearby genes, (2) their abundance, which may trigger aberrant meiosis through ectopic recombination, and (3) whether TEs are involved in the build-up of genetic conflicts and reproductive isolation.

The first aspect, insertional mutagenesis, is supported by many examples, such as disease-causing insertions in several organisms, including humans [83]. In general, there are less TEs inserting in coding regions than expected if insertions occurred at random [10,84]. TEs such as retrotransposons also contain promoter sequences that may alter the expression of genes at their vicinity (see Section 3.3). TEs can also trigger epigenetic silencing extending to genes nearby (e.g., [85]), or produce RNAs or proteins that induce damages. For example, competition of TEs with host genes for transcription factors [86] may lead to a loss in fitness. Another mechanism by which TEs may have a negative impact on their host’s fitness is ectopic recombination between non-allelic copies, which can lead to deleterious chromosomal rearrangements. Support for this scenario comes from the absence of full-length elements from highly recombining regions of genomes [84,87,88], and their accumulation in non-recombining regions [89,90,91]. Large families of TEs are more likely to be deleterious than smaller ones, since more aberrant pairings may occur during meiosis [10,72,92]. Heterozygous TEs are expected to be involved in ectopic recombination more often because of the lack of an allelic copy on the other chromosome [93]. Overall, this implies that the effects of ectopic recombination may be maximal for long, recently active TEs with many copies found at low frequency in populations. Finally, TEs may have an impact on the fitness of hybrids coming from divergent populations. The recent invasion of the *P*-element in *D. melanogaster* [12,13] and in *D. simulans* [9,94,95], which results in hybrid dysgenesis between individuals carrying the element or not is a classic example. Such mechanisms generate genomic conflicts and may promote speciation by reducing the fitness of hybrids.

This is not to say that TEs only have deleterious effects. TEs can also be recruited by positive selection, for example, because of their regulatory effects on neighbouring genes. This is exemplified by one of the most iconic cases of natural selection, the industrial melanism in peppered moths (*Biston betularia*), which is caused by a TE insertion at the *cortex* locus [96]. Cases of selective advantage have also been detected in *Drosophila*, where TE insertions are involved in resistance to insecticides [97] or response to oxidative stress [98], and in plants, where TE insertions are associated with adaptation to a broad range of environments [99,100,101]. However, it remains unclear how common adaptive insertions are. In a scan for adaptive TE in *D. melanogaster*, hundreds of putative adaptive insertions were identified [102] while similar population genetics approaches in *A. thaliana* [103] *D. suzukii* [104], human [105] and the lizard *A. carolinensis* [106] revealed much smaller numbers (less than 20) of positively selected insertions.

The likelihood for a TE to have an impact on fitness strongly depends on its intrinsic properties, such as insertion preference or transposition dynamics. These properties of TEs can be seen as the equivalent of life-history traits for species adapted to a specific ecological niche [107]. These niches often differ in their biochemical properties, such as DNA conformation, chromatin state or CpG content [108,109]. Some TE families [110] and endoviruses [108] are found more frequently near recombination hotspots, which may ultimately make them more deleterious if they engage in ectopic recombination. This might be due to a shared preference of recombination and transposition machineries for open chromatin [111]. In *Caenorhabditis elegans*, the cut-and-paste mechanism of transposition of DNA transposons may benefit from the double-stranded breaks that initiate recombination events, explaining their abundance near recombination hotspots [112]. Some TEs target specific DNA motifs, such as the ones found in *Drosophila* telomeres [113].

The abundance of TEs in a genome depends on the rate of transposition of progenitors, and the rate at which their copies become unable to transpose. This inactivation can be caused by the host’s control mechanisms or be due to incomplete duplication and truncation. These factors vary strongly across families and hosts. Moreover, the transposition rate varies over time, with waves of transposition associated with high activity followed by quieter periods, possibly due to the activation of the host’s control mechanisms [55,114,115,116,117]. These bursts produce cohorts of TEs having approximately the same age. Recent bursts of transposition may explain the excess of rare TEs observed in *Drosophila*, in association with purifying selection [118]. On the other hand, TEs produced by ancient bursts of transposition are generally at a much higher frequency or even fixed. This means that non-constant transposition may produce a signature in the TE frequency spectrum that mimic expectations under scenarios of purifying selection (recent burst) or balancing selection (ancient burst).

All the factors detailed above impact the host’s fitness, and the selective coefficient associated with each TE insertion, *s*. However, the efficiency of selection also depends on genetic drift, which becomes stronger as the effective population size *N_e_* decreases: if the product *N_e_ s* is lower than 1, the insertion will behave as if effectively neutral. The effective population size is highly variable across species and strongly depends on their demographic history [8,119,120]. Recent population bottlenecks reduce *N_e_* and may lower the efficiency of selection against TEs. In addition, demographic changes will shape the allele frequency spectrum of TEs [6]: reductions in *N_e_* should lead to a genome-wide excess of alleles at intermediate frequencies, while population expansions may lead to an excess of rare insertions. A complicating factor is that estimators of *N_e_* vary along the genome, due to the effects of selection at linked sites. Regions near a positively or negatively selected site display lower allelic diversity, and a higher rate of fixation of neutral and deleterious variants. This effect becomes stronger in regions of low recombination and is analogous to a local reduction in *N_e_* [121]. This leads to an accumulation of TEs at both ends of the frequency spectrum: more TEs will reach fixation in the region of low recombination than in regions of high recombination due to Hill-Robertson interference [122], but the remaining polymorphic TEs will also be at a lower frequency on average. In addition, since selection is less effective in regions of low recombination, they may accumulate TEs that are more deleterious, on average, than the ones inserting in regions of high recombination.

The interaction between selection, linked selection, transposition and preferential insertion has a strong influence on where TEs accumulate and reach high frequencies in the genome. It may explain the broad diversity of correlations between TE density or frequency and various genomic features. For example, in the green anole, simulations combining linked selection and preferential insertion in regions of high recombination reproduced the correlations observed between TE density, frequency, and recombination for near-neutral TEs [106]. While linked selection led to lower average TE frequency in regions of low recombination, preferential insertion led to a higher TE density in regions of high recombination, contrary to expectations under linked selection alone.

## 3. The Impact of TEs on Eukaryotic Genes and Genomes

### 3.1. The Impact of TEs on Structural Variation

TEs vary in their mechanisms and molecular processes of transposition, but they are unified by their capacity to produce structural variation. While definitions of structural variation have varied over time, contemporary definitions consider structural variation as insertions, deletions, inversions, or translocations, equal to or greater than 50 base pairs [123]. As the smallest active DNA transposons (MITEs) and retrotransposons (i.e., SINEs) are approximately 100 and 300 bp, respectively [33,124], TEs are structural variants. Depending on the mechanism of replication, they may produce additional structural variation at the site of insertion or the site of origin, in the case of cut-and-paste TEs. Most, if not all, TEs can also replicate host sequences [125], so structural variation arising from TEs can be diverse in origin, and due to the myriad of replicative mechanisms among TEs, diverse in form. While typified by insertion-deletion events, TE-associated structural variation commonly includes inversions and can be a source of copy number variations, large deletions, duplications, and translocations [126,127,128]. Beyond these basics, TE-associated structural variation is best understood in the context of specific TE types and their lifecycles.

Non-LTR retrotransposons can transduce their flanking DNA. Transduction of the 5′ flanking sequence can occur if the upstream flanking sequence contains an active promoter, which drives transcription and is eventually reverse-transcribed together with the element [129]. These cases are very rare. More common is the transduction of the 3′ flanking sequence due to the weak polyadenylation signal contained at the 3′ end of elements. In this case, transcription of the element continues until the next polyadenylation signal, taking with it the downstream sequence, which is reverse transcribed with the element [130]. The transduction of the 3′ flanking sequence, up to 1.6 Kbp long, is found associated with 20% of human *L1* elements [131,132]. LTR-retrotransposons can also mobilise non-TE DNA, but this typically occurs by transport of DNA (up to ~4 kb) captured between terminal repeats [125,133]. In addition, non-LTR retrotransposons can dramatically alter their site of insertion, resulting in structural variation. The insertion of an *L1* element is typically associated with the formation of a target site duplication, which, in some rare cases, can reach a few 100 bp in length [134]. The insertion of *L1* and *Alu* elements can also produce large deletions, up to a few Kbp, duplications or inter-chromosomal translocations [134,135,136,137,138]. Finally, non-LTR retrotransposons constitute a powerful source of microsatellites derived from the 3′ terminus of elements [139]. This phenomenon is particularly pronounced in some snakes whose genomes contain the highest density of microsatellites known in vertebrates [65]. These microsatellites are believed to be derived from the repetitive 3′ end of *CR1* LINE elements.

DNA transposons can give rise to a wide diversity of structural variation [33]. The classic “cut-and-paste” subclass of DNA transposons causes insertions at their target sites and can cause deletions or other anomalies through excision at the site of origin [140]—although many likely regenerate at the donor site by double-stranded-break-induced homologous recombination and concordant gene conversion to eliminate the deletion site at the origin [141,142]. “Cut-and-paste” transposons can also capture host DNA sequences between their terminal inverted repeats during transposition, and can generally exceed retroelements in terms of maximum transported bases, with some types being capable of relocating 10–20 kb of additional host DNA [125]. In rice, it was shown that a large number of genic fragments were mobilised and amplified by the *Pack-MULE* DNA transposon [143]. *Helitrons* use a rolling-circle replication [144], contingent upon a single-stranded DNA break and excision at the origin site. This would seem to exclude deletion variants at the origin site—as the single strand is typically replaced—but creates a seemingly unrivalled capacity for replication of host DNA sequences downstream from the origin site, although this is typically limited to a maximum of a few Kbp [125,145]. The *Mavericks*/*Polintons* [37,38] subclass are very large DNA transposons that are typically around 20 kb and can reach 40 kb. The capacity of their independent DNA polymerases to perform long-range amplification of up to approximately 45 kb allows them to potentially move very large fragments of host DNA along with them [125].

While the movement and replication of TEs is an obvious source of structural variation, TEs can cause structural variation through several indirect processes, particularly those related to DNA repair and recombination [75,126,146,147]. These processes can have a profound impact on genomic stability but can also be an important source of evolutionary novelty, for instance by mediating segmental duplications [148]. TEs can drive ectopic recombination events when similar TE sequences align in non-homologous locations of the genome. When this occurs on the same chromosome it can cause major chromosomal rearrangements, such as large inversions [149,150], and when it occurs between different chromosomes it can cause translocations. Ectopic recombination events between closely located TEs can also cause any number of large localised structural variants [126]. For example, if two nearby TEs of the same orientation align, this can cause a reciprocal deletion on one chromatid and duplication on the other. If the repeats are in inverted orientation this can cause an inversion of the flanked sequences. Non-reciprocal crossover between non-homologous repeats can likewise result in large uncoupled deletions or duplications. Similar processes can occur when the end-joining of broken DNA occurs between non-homologous regions through single-stranded annealing or microhomology-mediated end-joining of shared repetitive sequences [147]. TEs also directly cause strand breaks during insertion [75] and excision, and can indirectly cause strand breaks during DNA replication due to secondary structures formed during DNA replication [127]; these breaks can be associated with recombination and homologous end-joining associated duplication and deletion events [146].

### 3.2. The Impact of TEs on the Genomic Landscape

As a consequence of their effect on fitness, TEs are not uniformly distributed in genomes and their accumulation in some regions and their absence from others has a defining impact on genomic landscapes. Not surprisingly, there is a general tendency for TEs to accumulate in gene-poor and low recombining regions because their negative effect is reduced there (see Section 2.3) [60,89,151,152,153,154]. As the negative effect of TEs is often length-dependent, long elements, in particular, will accumulate in low or non-recombining regions [89,91]. The long term accumulation of TEs in gene-poor regions will eventually increase the length of intergenic regions and overall physical distances within genomes. This process is exemplified in human where long stretches of intergenic sequences are composed almost exclusively of TE sequences, mostly from the *L1* family, while other regions, such as the HoxD gene cluster and recombination hotspots are almost completely devoid of TEs [60,88]. Interestingly, *Alu* elements, which are SINEs relying on *L1* for their mobility, have a different genomic distribution than *L1* and tend to be distributed in genic regions [154], possibly because of an insertion bias or selective processes. The clustering of TE-derived sequences in a specific genomic location can result in the evolution of satellites, as exemplified by the accumulation of sequences derived from the *Bari* DNA transposon in *D. melanogaster* [155].

The accumulation of TEs can potentially affect the regional base composition of genomes since TEs often harbour base compositions that are substantially different from the genome average [156,157,158]. In lizard and fish, for instance, the GC content of non-LTR retrotransposons ranges from 33% to 55% while the genome average is ~41% [158]. Even larger differences were reported when comparing class I and class II elements in fish [159]. An accumulation of AT-rich TEs, such as the mammalian *L1*, in a specific region of the genome, could have a substantial effect on the heterogeneity in base composition and could contribute to the isochoric organisation of some vertebrate genomes, although the main determinant of base composition heterogeneity is biased gene conversion [160]. In fungi of the genus *Leptosphaeria*, the differential amplification of AT-rich TEs resulted in substantial differences in the overall genomic base composition among strains, with ~45% GC in strains that have experienced TE amplification and 51% in strains that have not [51].

Although the recombination rate is one of the main determinants of TE distribution in genomes, the interplay between TEs and recombination is quite intricate [161]. If the local recombination rate determines the probability of fixation of a TE, TEs in turn can modify the recombination rate. TE activity tends to be deleterious to the host and is thus repressed epigenetically. TE repression can cause chromatin compaction, which in turn represses recombination since recombination initiation more often occurs in an open chromatin state [160]. This model is supported by the correlation between the methylation status of chromatin and recombination rate [161,162,163], by the experimental disruption of methylation that results in increased recombination [164,165] and by the repression of recombination triggered by specific insertions [166,167]. The reduced recombination rate caused by TEs can eventually affect the dynamics of linked alleles in the genome and a recent study proposed that polymorphic TEs increase linkage disequilibrium and consequently the size of chromosomal blocks subject to selection [168]. In some other cases, it was shown that TEs can increase the recombination rate locally, as was observed in the vicinity of *Alu* elements in human [169], possibly because *Alu* sequences contain recombinogenic motifs.

### 3.3. The Impact of TEs on Gene Regulation

TEs have been shown to influence patterns of gene expression in multiple ways and scientists are just beginning to appreciate the considerable impact TE-derived sequences have had on the regulation of host genes [2,3,170,171,172,173]. Although most studies in this field come from model organisms, TEs likely constitute the main source of regulatory novelty in some lineages such as primates [174,175]. To maintain their ability to move around the genome, TEs encode intrinsic regulatory sequences necessary for their own transcription. These sequences can, in turn, affect the expression of host genes. In addition, regulatory elements may evolve by exaptation, i.e., the evolution of a new function out of non-adaptive material [176]. This can occur when TE-derived sequences accumulate mutations, which can eventually evolve into a regulatory element. A number of studies in the past two decades have demonstrated that TEs constitute an important source of *cis*-regulatory motifs in the form of transcription factor binding sites (TFBS), distal enhancer, suppressor or insulator. For instance, a substantial fraction of TFBS in mammalian genomes was shown to be derived from TEs [177,178,179]. These include binding sites for TFs implicated in immune response such as *STAT1* [180] or pregnancy such as *cAMP* [181]. In addition to a direct effect on genes, some TFBS generated by TEs can affect the three-dimensional organization of chromatin and thus participate in the regulation of genes that are not in close proximity. This process is exemplified in mammals where many of the *CTCF* TFBS, which play an important role in the definition of topologically associated domains (TADs), are derived from TEs [182,183]. A similar effect of TEs on the spatial organization of the genome is found in plants where miniature inverted-repeat transposable elements (MITEs) are associated in rice and sorghum with the presence of matrix attachment regions, which serve as anchors for loops [184].

The spread of TFBS tends to be lineage-specific and thus promotes regulatory divergence among lineages [177], but the invasion of different eukaryotic lineages by the same horizontally-transferred TE can also result in the independent spread of the same TFBS among distantly related organisms [185]. In some cases, TEs can contribute directly to the core promoter sequence of genes and replace pre-existing promoters [186,187,188,189,190]. Bioinformatic and experimental evidence have now demonstrated a direct effect of TE-derived regulatory sequences on a vast number of genes, such as *POMC*, an important brain-expressed gene which controls food intake in human [191]; *Agouti*, a colour-coat gene in mice [192]; *ZmCCT9*, a transcription factor regulating flowering time in *Z. mays* [99] and *Zmr1*, a gene that controls melanisation in *Zymoseptoria tritici*, a trait that affects the survival of this fungus under stressful conditions [193]. It should be noted however that, although substantial, the recruitment of TEs in gene regulation remains a rare occurrence and there is a tendency for TEs to be under-represented upstream of genes [194]. The impact of TEs on gene regulation also differs considerably among TE categories. For instance, SINEs and LTR retrotransposons seem to have been recruited disproportionately as gene regulators in mammals, compared to non-LTR retrotransposons [2,195,196].

The effect of TEs on gene regulation may be particularly significant in the evolution of complex functions that require the concerted regulation of multiple genes interconnected in a regulatory network. A gene regulatory network is a framework of interactions between molecular regulators (e.g., transcription factors) and substrates (e.g., transcription factors binding sites) that orchestrate the expression of genes involved in complex biological processes [197]. It has been suggested that the evolution of gene regulatory networks can be induced by a wave of TEs invading and amplifying within a eukaryotic lineage [3,198]. This suggestion is based on the observed associations between expansions of specific TE families and the dispersal of regulatory elements, which coincides with major evolutionary events, such as the emergence of mammalian pregnancy [181], and the evolution of the mammalian neocortex [199]. It remains possible that insertion bias or selection favours the retention of specific TEs in genes that share similar profiles of expression but that the TEs have a minimal impact on the expression of those genes. However, there is a growing number of studies demonstrating a direct role of TEs in the evolution of gene regulatory networks [3,178] which are involved in important biological processes such as pluripotency [200], innate immunity [180], placentation [181,201], early mammalian embryonic development [202], and dosage compensation on sex chromosomes [203]. In *A. thaliana* and in *Z. mays*, the activation of stress-sensitive TEs affects the expression of nearby genes and may be involved in the evolution of stress-response regulatory networks [204,205]. Not only are TEs involved in the ’wiring’ of connections between genes but they can also participate in the ’re-wiring, and thus the evolution, of these regulatory networks [178,206,207]. One of the mechanisms that enable TEs to rewire regulatory networks could be through non-allelic gene conversion between TE copies [208,209], which could aid the spread of beneficial mutations, and allows selection to improve the functionality of the network [208,210]. As a mechanism, non-allelic gene conversion between copies of TEs is suggested to allow a faster share of beneficial mutations across different TE copies than the whole rewiring process by transposition alone [208].

There are many other ways TE insertions can affect the expression of genes, independently from their ability to provide specific regulatory motifs. TEs inserted in introns can reduce the efficacy of transcription by inserting premature polyadenylation sites or because the unusual base composition of the TE sequence reduces the amount of transcript being produced, as demonstrated for the A–rich L1 element of mammals [211]. TEs can also introduce splicing sites that can result in the production of incomplete or chimeric transcripts [212]. These effects are reflected in the biased orientation of intronic TEs which tend to be in the orientation that has the least negative effect on the transcription of the gene they are inserted in [154,213]. TEs inserted in the untranslated regions (UTRs) of genes can also have a profound impact on their regulation. For instance, a *POGON1* DNA transposon insertion in the 3′UTR of the *CG11699* gene in *D. melanogaster* produces a shorter 3′UTR which is adaptive and confers resistance to xenobiotic stress [214]. Another example is found in the flowering plant *Capsella rubella* where a *Helitron* insertion in the 3′UTR of the *FLC* gene affects mRNA stability and result in a reduced expression level of *FLC* [215]. In mouse and human, the presence of SINEs in the 3′UTR of a gene can result in the formation of a STAU1 binding site and target the mRNA to *Staufen*-mediated decay, thus downregulating expression [216,217]. Finally, TEs can play a role in gene regulation in *Trans* by participating in the evolution of regulatory non-coding RNAs (such as miRNA, piRNA or long non-coding RNAs) [2,218]. A number of miRNAs are derived from TEs (~20% in the human genome [199]) and TE-derived sequences confer the functionality of different types of small and long non-coding RNAs [2].

An indirect way TEs can affect the regulation of genes results from their epigenetic repression by the host. The activity of TEs is deleterious in general and a number of mechanisms have evolved to silence TEs in eukaryotes, and one of the known strategies for silencing TEs is performed at the epigenetic level [219,220,221,222]. Epigenetic silencing of TEs can be performed through different mechanisms, including DNA methylation, which is arguably the most widely used strategy by higher eukaryotes for repressing TEs [223,224], the KRAB-zinc finger proteins (KZFPs) [225], and RNA interference by PIWI-interacting RNAs (piRNAs) or small interfering RNAs (siRNAs) [226]. These different mechanisms can repress TEs but can also affect host genes. This effect is more pronounced if TEs are at a close distance from host genes, but epigenetic repressive marks on TEs can extend beyond 20 kb from a repressed TE insertion [227]. In *A. thaliana* the repression of TEs by DNA methylation extends 500 base pairs on each side of the element [228], and in many cases can affect neighbouring genes [229,230,231,232]. In mice and *Drosophila*, enrichment level with the repressive H3K9me2 mark decreases with increased distance from polymorphic TE insertions [227,233]. Since the spreading of repressive marks from the selected TE insertions may influence the expression of nearby genes, it is likely to influence the fitness of the host [234,235]. It is thus expected that TEs with repressive marks acting on functional genes will be deleterious, and therefore would be eliminated by purifying selection. Interestingly, genome-wide population studies of TEs in plants show consistency with this prediction, as unmethylated TEs are usually closer to genes than methylated TEs [234,236]. Moreover, studies that examined population frequencies of TEs reported that low-frequency TEs are more likely to be TEs with higher enrichment of repressive mark at neighbouring genes [227,229], suggesting that these TEs are more strongly selected against. The unintended consequence of the epigenetic repression of TEs led to the intriguing idea that epigenetic repression is a “double-edged sword”, which is beneficial to the host as a defence mechanism but also has a cost since the repression of TEs will affect gene expression [221].

### 3.4. The Impact of TEs on Protein-Coding Sequence

TE-derived sequences can also significantly impact the protein-coding capabilities of eukaryotic genomes, either by providing motifs that can become embedded in the protein-coding sequence of host genes, by becoming a protein-coding gene through a process called domestication or by mediating the duplication of host genes. TE activity may lead to the development of evolutionary novelties through gene fusion events—phenomena, in which a single transcriptional unit, known as a chimeric gene, originates from different source genes [237,238]. Several TE-mediated fusion mechanisms have been proposed, including gene capture by LTR-retrotransposons or DNA transposons (*Pac-MULEs*, *Helitrons*), retroposition, and reverse end transposition [237,239]. These mechanisms can create novel sequence combinations through the juxtaposition of exons from different genes (i.e., exon shuffling) or insertion of retrocopies within introns of existing genes. Numerous examples of chimeric genes resulting from the activity of TEs have been identified in a variety of eukaryotes. In a recent study, 94 cases of fusion genes containing a transposase motif were discovered in vertebrates [240] demonstrating that this mechanism of gene evolution is relatively common. Interesting examples of fusions include *TRIM5-CypA*, an antiviral gene that evolved independently in New and Old World monkeys [241,242,243], the primate *SETMAR* gene that plays a role in histone methylation and DNA repair [244,245] and the *KRABINER* transcription factor in bats [240]. The *KRABINER* example is particularly telling because it illustrates the features of the transposases that make it particularly prone to recruitment in a function that necessitates recognition and binding of specific DNA motifs [240].

Under certain circumstances, the host may harness parts or whole sequences of TEs and repurpose them for its own benefits in a process known as molecular domestication. Genome-wide studies documented numerous examples of functionally important non-coding [218,246] and coding sequences that had been co-opted from a variety of TEs [247,248], including transposases, the critical enzymes used by DNA transposons for their mobility. One of the most iconic examples of transposase domestication is the case of the recombination-activating genes (RAGs), which play a pivotal role in V(D)J somatic recombination, a fundamental element of the adaptive immune response of jawed vertebrates [249]. Guided by the localization of recombination signal sequences (RSSs), *RAG1* and *RAG2* facilitate the separation, rearrangement, and rejoining of DNA segments in numerous permutations, creating a diverse reservoir of T- and B-cell antibodies. While the origin of *RAG1* has been traced to transposases encoded by the *Transib* family of DNA transposons [250,251], the origin of *RAG2* remained uncertain until the recent discovery of an active transposase in the genome of the lancelet, which contains both *RAG1*- and *RAG2*-like genes in the correct orientation flanked by terminal inverted repeats (TIRs) that resemble the RSSs [251,252]. Another famous example of transposase domestication comes from plants with the *FAR1* and *PHY3* genes, which are transcription factors that modulate light signalling [253]. Interestingly, independent domestication of transposases derived from the same TEs can result in the convergent evolution of genes with similar function. This is the case of centromere-binding proteins in fission yeast and mammals that have convergently evolved from a transposase encoded by a *pogo* element [254]. Domestication is not limited to DNA transposons and numerous co-options of retrotransposon-derived sequences have been documented. The domestication of the *L1* reverse transcriptase has been documented as exemplified by the *TERT* gene, which plays a role in the maintenance of telomere length [255]. Numerous genes have been classified as derivatives of proteases, integrases, *pol*, *gag* and *env* derived from LTR retrotransposons [247]. Several of these products have been linked to the formation and function of the placenta, a transient organ facilitating interactions between the mother and the developing foetus. *Env*-derived *syncytins* have been independently co-opted numerous times across the mammalian tree of life, with homologs described in both eutherians [256,257,258] and marsupials [259]. In rodents, certain *syncytins* play a pivotal role in the formation of the placenta, mediating the fusion of maternal and fetal trophoblasts [260]. Furthermore, some of them are known to possess immunosuppressive properties, which are hypothesised to play a role in defending the foetus from the maternal immune system [261].

The examples of molecular domestication cited above resulted from the modification of TE-derived protein-coding sequences from TEs for a new purpose. However, in some rare cases, the transposition activity itself has been domesticated such as in the case of *Drosophila*, where TEs are involved in facilitating the elongation of telomeres at chromosome ends. Since chromosome ends may lose nucleotides with every DNA replication cycle (also known as the “end replication problem”), organisms have evolved various mechanisms to prevent the shortening of chromosomes and retain their telomeres. Several eukaryotic organisms use the telomerase enzyme, which counteracts chromosome erosion by promoting the synthesis of repetitive sequences to the ends of chromosomes [262]. In contrast, some *Drosophila* species like *D. melanogaster*, have recruited specialised transposable elements from the *Jockey* subfamily (*HeT-A*, *TART* and *TAHRE* families) to protect their telomeres by transposing to the ends of the chromosomes [263,264,265,266]. While these retrotransposons appear to have evolved in a symbiotic manner, another hypothesis emphasises the selfish nature of these TEs, suggesting that they selfishly over-replicate in these genomes [267]. This over-replication may lead to a diversification of the specialised TEs and an eventual disappearance in species such as *Drosophila biarmipes* [266]. Another example of domestication with a direct functional impact on chromosomal evolution is the role of TEs in maintaining the stability of eukaryotic centromeres and the silencing of heterochromatin [267]. For instance, the genome of the social amoeba *Dictyostelium discoideum*, whose centromere is rich in TEs (86%, 171–361 bp), is dominated by LTR retrotransposons [268]. The centromere and the surrounding region containing the tRNA genes appear to harbour several copies of these TEs, which have a strong insertional preference for the region as a result of low mutagenesis and the presence of relatively safe integration sites [269]. This hypothesised domestication of TEs is thought to be involved in a complex control network that comprises LTR retrotransposon families (*Skipper* and *DGLT-A*), the RNAi machinery, and centromeric histones that regulate retrotransposition and kinetochore assembly [269]. Thus, TEs in the centromere of *D. discoideum* may have co-evolved with its host to provide the necessary substrate for kinetochore complex formation in return for the ability to accumulate in the genome near “low-danger” tRNA genes [270].

Another way that TEs can contribute to the emergence of new genes is by mediating the duplication of host genes. The ‘birth’ of a gene is associated with a duplication event, in which a gene copy emerges as a result of replication (DNA-mediated) or reverse transcription (RNA-mediated) [237,271]. In the latter process, known as retroposition, the enzymatic machinery of active retrotransposons or retroviruses orchestrates the reverse transcription of an mRNA transcript and its integration into the genome, leading to the emergence of a retrocopy (see [271,272] for mechanisms). Retrocopies are intron-less, may contain polyA tails at the 3′ end, and, in the case of LTR-mediated retroposition, flanking LTR regions [239,271]. Due to the initial bias in sequencing primarily mammalian genomes, early studies of retroposition focused on understanding the role of non-LTR retrotransposons in mediating the process. While the contribution of some members of this group, particularly *L1* elements, is well-understood [273], there is growing evidence of the occurrence of LTR-mediated retroposition, with evidence from plants [274] and animals [275]. Importantly, not all non-LTR and LTR retrotransposons are capable of effective retroposition, as evident by the example of *CR1*s, the predominant retroelements in bird genomes, which are generally unable to generate retrocopies [276]. There are several evolutionary pathways that can be followed by a retroposed gene copy. The vast majority of retrocopies exist in the form of retropseudogenes [212], non-functional relics of the parental gene that had acquired frameshift mutations and premature stop codons [237]. While pseudogenization is commonly associated with loss of function, it does not necessarily represent a “dead-end” for a gene. There is growing evidence that neutrally-decaying retropseudogenes and non-functional retrocopies may serve as components of functionally important non-coding RNAs, such as miRNAs [277], siRNAs [278] and lncRNAs [218]. However, some retrocopies can maintain their protein-coding abilities and are referred to as retrogenes. This process has led to the increase in the copy number of ribosomal protein genes in fission yeast, which may improve glucose consumption rate through enhanced ribosome biogenesis [279]. Another interesting example of retrocopy evolution is represented by the cell-cycle gene *CDC14B*, which encodes a protein that stabilises microtubules, and its ape-specific retrocopy dubbed *CDC14Bretro* [280]. In the African ape lineage (gorilla-chimpanzee-human), the protein product of the retrocopy has been found to occur in association with the endoplasmic reticulum, indicating acquisition of a novel function, while in the Asian ape lineage (orangutan and gibbon), the retrogene was found to associate with microtubules, similar to the original copy of *CDC14B* [280]. This retention of the parental gene function represents another scenario of retrogene evolution, which occurs if the increased expression of the encoded product provides a fitness advantage or the functionality of the parental gene is lost due to pseudogenization or deletion. The latter case leads to the emergence of the so-called “orphan” retrogenes, which have been identified in several studies [281,282].

## 4. The Role of TEs in the Diversification of Life

The previous sections of this review have described the numerous mechanisms by which TEs may impact their host genomes. Either through exaptation or positive selection on new insertions, TEs have been an important source of evolutionary novelties and have been instrumental in the evolution of biological processes that have affected the diversification of eukaryotes [283]. For instance, many of the traits that are responsible for the evolutionary success of mammals have been profoundly impacted by TEs, from placenta formation [182,202] to the evolution of a complex brain [199,284]. TEs could also affect the diversification of life by directly driving the formation of species [285,286]. The role of TEs in speciation remains controversial and it is unclear to what extent TEs actually contribute to the processes that lead to reproductive isolation. A role of TEs in speciation is supported by the correlation between speciation and bursts of TE amplification. In a number of taxa, including rodents [117,287], bats [288], and woodpeckers [55], rapid speciation correlates with the amplification of TEs, leading to the question of whether one causes the other. Because TEs amplify in bursts and could thus trigger species formation, as well as the evolution of novel phenotypes, over a short period of time, TE amplification could provide an explanation for the observation that evolution often proceeds in jumps (at the time of a burst of TE amplification) followed by a period of stasis (when TE activity is low), a model called the “punctuated equilibrium” theory of evolution [289].

Although the correlation between the rate of speciation and transposition seems solid, at least in some lineages [290], it remains a correlation and it does not, in itself, provide a mechanism. In fact, this observation is consistent both with the idea that TEs are drivers of speciation [291,292] but also with the view that TE amplification is a consequence of the speciation process [293]. The “TE thrust” hypothesis [291] proposes that through active (transposition and integration) or passive (ectopic recombination) thrust, TEs can cause significant structural changes in the genome, allowing the development of phenotypic diversity, which enables populations to adapt and occupy new ecological niches. Consider the event of a bottleneck in the presence of abiotic differences, where effective population size is low, and there is a loss of genetic variation due to the founder effect. In line with the genomic shock model proposed by McClintock [294], TEs may mobilise in response to environmental stress, possibly because of disruption of epigenetic repression [285,292], and allow an increase in genetic diversity, with neutral or slightly deleterious TEs actively transposing and becoming fixed in the genome. Abiotic differences may drive ecological speciation and the diversification of lineages, with TE thrust playing a key role in the realization of adaptive potential [291]. The TE thrust hypothesis has been invoked to explain the difference in diversity of gymnosperms and angiosperms [63]. Angiosperms have younger and more active TE families in comparison with extant gymnosperms and this could explain the lack of evolutionary innovations and speciation in gymnosperms. The TE Thrust hypothesis, as well as the epi-transposon hypothesis [292] present TEs as drivers of speciation, suggesting a causative role of TEs in achieving speciation.

However, the carrier subpopulation (CASP) hypothesis argues from a neutralist perspective [293]. It proposes that TE amplification may be a byproduct of speciation when populations subdivide, leading to the random assortment, fixation and amplification of TEs due to genetic drift. Thus, neutral or slightly deleterious active TE families may have a large impact on genome evolution if they amplify into larger families, thereby resulting in TE-mediated diversification, which may further lead to speciation by increasing the chances of reproductive isolation. This hypothesis implies that the TE landscape of a species may be indicative of structural changes in populations over time and may reflect recent or ongoing subdivisions in the population that may be associated with speciation events. We caution here that these hypotheses are not mutually exclusive—TEs may be largely drifters accompanying demes that result in new species, but at the same time, some TEs may have properties that are adaptive and may drive ecological speciation by allowing populations to evolve independently.

Another indication that TEs may play a role in speciation comes from their potential ability to promote the evolution of reproductive isolation. TEs can mediate genomic divergence between nascent species, for instance by causing chromosomal rearrangements [295], which could result in hybrid inviability or sterility [296,297]. Another possibility is an increased activity of TEs in hybrids [298,299,300,301]. Since the early discovery of hybrid dysgenesis in *Drosophila* [13,14,15], the uncontrolled amplification of TEs in hybrids between populations or species that differ in their ability to regulate specific transposable elements provides an appealing mechanism for the evolution of post-zygotic reproductive isolation. The disruption of the epigenetic repression of TEs observed in some crosses could certainly result in reduced viability or fertility in the hybrid [285], yet the number of documented cases remains very low and several known examples involve artificial crosses between relatively distant species [302,303]. In fact, recent hybrids between incipient yeast species [304] or between well-differentiated species [304,305] have failed to detect any evidence of TE de-repression. Thus the role of TEs in mediating post-zygotic isolation remains unclear and will require further studies [306].

## 5. Conclusions

The goal of this review was to emphasise the considerable number of ways in which TEs can impact the genome of their host. With the exponential increase of genomic data and improvement of annotation methods, researchers will likely discover new ways by which eukaryotic genomes have been affected by TEs. There are however a number of questions that remained open and that will benefit from the use of novel analytical and experimental approaches. Although TEs are subject to the same evolutionary forces as any type of mutation, the range of effects produced by TEs likely differ and will require further investigation. Overall, further work is needed to quantify the distribution of fitness effects of TEs relative to other types of variation. In particular, the proportion of TEs that are under the effect of selection may vary substantially, depending on their intrinsic properties and their host’s dynamics. Future work may gain from the study of the distribution of fitness effect of coding variation [307], which will provide a theoretical framework upon which models incorporating TE specificities could be built. Such models including important aspects of TE biology will certainly benefit from the development of fast and efficient simulators (e.g., [308]), but also functional studies in laboratory models. Genomics analyses have generated many hypotheses about the functional impact of TEs that are still in need of experimental validation. Approaches that link the distribution of TEs with functional analyses are particularly promising [309] and the increasing use of the CRISPR-Cas9 technology should provide experimental validation on the impact of specific TE insertions [180,310,311].

## Data Availability

Not applicable.
